# Hope of Patient Recovery in the ICU From the Viewpoint of Iranian Nurses: Concept Analysis

**DOI:** 10.5539/gjhs.v6n6p105

**Published:** 2014-07-15

**Authors:** Mojgan Jahantigh, Nasrin Rezaee, Nahid Rezaei

**Affiliations:** 1 Department of Nursing, Zahedan University of Medical Sciences, Zahedan, Iran; 2 Pregnancy Health Research Center, Zahedan, Iran; 3 Department of Nursing, Tehran University of Medical Sciences, Tehran, Iran; 4 Zahedan University of Medical Sciences, Zahedan, Iran

**Keywords:** hope, concept analysis, nurse, ICU

## Abstract

Nurses’ care quality for patients in the ICU depends on their degree/level of hope to improving patient, but there is no consensus on the concept “hoping to improve patient.” The purpose of the present study is to analyze the concept nurses hoping to improving patient in the ICU. To analyze this concept, hybrid model is used which consists of theoretical phase, field work phase, and final analytical phase. In field phase work, semi-structured, face to face and individual interviews were done for nurses working in the ICU, and the data gathered from the interviews were analyzed using inductive content analysis. In theoretical phase, the concept hoping to improve patient was characterized by being available, being professional, expecting positively, and being future- oriented. The scientific definition of this concept was explained through properties which are necessary for qualified nursing care. In field work phase, the categories include nursing care, inner feeling, belief and consequences. In final analytical phase, final definition of the concept was explained through properties such as dynamic expectation, being realistic, and being goal- oriented which is a better function and attitude in effective nursing care accompanying peace of mind for nurses. Concept analysis showed that nurse’s awareness of hoping to improve patient helps the nurse do his job in the best way and with peace of mind.

## 1. Introduction

Despite the extensive and expensive treatment or health care services offered to patients in the ICU, many shortcomings in offering health care services to these patients are reported. Fundamental changes and offering new strategies seems quite necessary. These strategies must improve the quality of offering health care services to patients and satisfy the relatives of the patient in the way the services are offered. The empirical evidence shows that nurses hoping to improve patient in the ICU try more, and nurses, who take care of patients under any physical circumstances, perform more effective nursing care.

In nursing theories, Meleis (2011) believes that partial care is taken into consideration in nursing concept. She says, in Benner theory, that care is the essence of the nursing. Leininger assumes care to be public, and it varies from one culture to another. In Watson’s view, care is the moral perfection. Orlando also defines nursing as interacting with the patient to satisfy his immediate needs (Meleis, 2011). Nursing position includes person’s behavior, nurse’s reaction and nursing action appropriate to one’s needs. The nurse is responsible for the person receiving care. Considering nursing definition whose focus is on the interpersonal interaction between the nurse and the patient, one of the concepts affecting nurse’s reaction and appropriate nursing action is the hope concept in nursing. Hope is an abstract concept and is defined in different textures, but hope in nursing is mainly concentrated on patients such as those suffering from cancers, HIV and on carers’ patient’s family or other concepts such as life quality (Kylmä et al., 2009; Rustøen, 1995). The hope concept is less focused from the viewpoint of nurses; therefore, there are lots of ambiguities in understanding it in nursing context which necessitates the use of concept analysis. Concept analysis is a precious method to clarify concepts which are frequently and ambiguously used in nursing. The purpose of concept analysis is to identify and search concept properties in order to clarify and explain the meaning and nature of the concept in nursing (Haase et al., 1992). In other words, concept analysis is to generally separate the discussing phenomenon with the prospect of giving us a deeper appreciation of quality at the bedside (Tutton et al., 2009). Since it seems that the hope concept can affect how to care, and less quantitative study is done in Iran, and no study is done on analyzing the hope concept, analyzing the hope concept in improving patient is picked to be scrutinized. Iran is an ancient country located in the Middle East with more than 5000 years of civilization and a life expectancy of 71 years. Unfortunately, the wrong cultural beliefs about critical care are still present among people indicating that the hoping to improving patient in the ICU taboo has not yet broken and many Iranians believe that hospitalizing in ICU is equivalent to death and end of life (Borimnejad et al., 2014). In addition, the history of critical nursing in Iran does not reflect the same background as that of Western countries.

### 1.1 Aim

The aim of this study is clarifying and better understanding the hope concept in nurses working in the ICU.

## 2. Method

In this study, hybrid model is used to analyze the concept “nurses hoping to improve patient in the ICU. The hybrid model is based on developing concept through qualitative analysis of the phenomena in the way they occur. In hybrid model, an approach, which can mix theoretical and experimental surveys and can purify high- frequency concepts, is used to complete the concept since this concept analysis model extracts knowledge and awareness in clinical affairs, it can prove helpful in studying important and fundamental phenomena in nursing. Hybrid model includes three phases: theoretical, field work, and final analytical phase (Schwartz-Barcott & Kim, 2000).

### 2.1 Theoretical Phase

To review studies in this phase, articles published in “Nurse’s hoping to improve patient” were sought using websites such as Google Scholar, Pre Quest, Science Direct, and Pub Med with key words “Hope” and “Nurse” in the title and abstract from 1990 to 2011. To search the articles, manual search method is used, too. In reviewing of studies, all the articles with the above mentioned keywords were first sought. Then, articles focusing on the patient hope concept or his caring were selected. Among other articles, the emphasis was on the qualitative approach. If there were an access to the full article, it would be reviewed and studied.

#### 2.1.1 Concept Definition

Hope in is defined in Oxford (2010) as “wanting to realize what is possible” and in Webster dictionary (1995) as “feeling what you want to happen”. In reviewing of the studies done, hope is to believe a better feeling in future and is remembered as a concept useful for having a healthy life. In fact, hope is a necessary factor in every dimension of life, and with its penetrating power system to gain new experiences and to generate new forces in the person (Ghahremani et al., 2006). Besides, hope is a response to a threat which leads to achieving aims (Klotz, 2010). Gebhardt et al. (2011) quote from Stotland that hope is much expectation for success and despair is little expectation for success. In Synder’s view (1995), hope is not a passive excitement appearing only in dark moments of life, but it is a cognitive process with which people actively follow their aims. In his belief, hope is a process with which people determine their aims, create strategies to achieve them, define motivations to perform theses strategies to achieve them, define motivations to perform these strategies, and to sustain them along the way.

Reviewing studies done on health shows that hope is more taken into consideration as a concept from the viewpoint of patients and their relatives careers. Also, with instrument development for this concept, quantitative studies like hope in cancer patients, psychopathic patients and the elderly are done. In this regard, hope and life quality in cancer patients showed that hope has a positive relationship with preserving life quality in these patients. Other results of the mentioned study also showed that coping with life quality experience is of high importance, and hope is one of the coping strategies. As a result, preserving or improving life quality is one of the vital roles of the nurses (Rustøen, 1995).

In nursing, hope is an important concept. Nurses are capable of creating hope in patients or help them keep on with a little hope they have. That is why researchers try to define it, try to help the patients, and the staff comprehends it and applies it in their daily experiences of life (Klotz, 2010). In Tutton’s study (2009), hope has been defined as a part of patient’s improvement, and nurses are believed to play a major role in making patients and their families hopeful. In addition, hope is a dynamic concept looking into the future. In nursing, hope covers a range from illness to improvement which is often a psychological process helping people go beyond their problems and have a positive attitude toward the future (Klotz, 2010).

Kylma et al. (2009) declared that hope is important for both living and dying. It is noteworthy that hope is a part of every relation between a nurse and a patient. A hope recognized by the nurse is not only a value but also necessary for health (O’Connor, 1996). With all the above definitions, Fitzgerald Miller (2007) in defining hope believes that the meaning and importance of hope depends on the life situation and personal philosophy of each individual.

#### 2.1.2 Review of Studies

Reviewing the studies done on hope concept can help clarify this concept and its specifications. The summary of the studies done on this concept, antecedents and consequences is presented in Table 1.

**Table 1:** Some studies done on the concept

Author	Field of study	Attributes	Antecedents	Consequences
Tutton (2009)	Hope concept discovery as a concept in nursing	- Expectation - Attaining the goal	- Part of the cure - Inner power - Positive attitude	- Helping patient improve - Identifying effective supportive mechanisms
Koltz (2010)	Hope in nursing interventions for patients		- Nurse active participation - Professional skills	- Relaxation - Security - Repose
Gillespie et al. (2007)	Flexibility concept analysis	-Self efficacy - Hope - Compatibility		- Coping with adversity
Benzein (1998)	Hope concept analysis	- Future oriented - Positive attitude - Activity - Realism - Purposefulness - Interpersonal Relations	- Stress - lack - life threatening situation - Provoking frustration	- Compatibility - Regaining - New strategy - Calmness - Promoting life quality - Physical health
Gebhardt (2011)	- Brain injury - Nurse’s hope and patient care for his improvement		-Braine injury	- Getting Better
Moke et al. (2010)	Hope perspectives of health workers		- Despair	- Hope

#### 2.1.3 Concept Attributes

In the final section of the theoretical phase, the attributes of the concept are clarified. Attributes are aspects of the concept repeating again and again while defining or explaining the concept. Without attributes, no concept analysis is feasible. These attributes are classified to present a clear picture of the concept. The attributes of this concept which are implicitly mentioned in the results of the studies are presented.

Reviewing Literature, hope is known as a strategy for compatibility and also a factor promoting patients life quality. Expectation, mobility, realism, goal- oriented, trying to improve, peace and cognitive process are a few to name of hope attributes (Benzein & Saveman, 1998; Mok et al., 2010). It is generally said that hope in nurses is a group of features necessary for promoting patient’s health using care quality.

### 2.2 Field Work Phase

Field work phase started with data gathering. In this phase, data are gathered through semi- structured, face to face and individual interviews.

#### 2.2.1 Sample

Due to the fact that 6 participants can be chosen in conceptualization (Schwartz-Barcott & Kim, 2000), the interview started with 7 nurses working in the ICU of the two educational hospitals in Tehran. The participants include 5 women and 2 men whose age range was between 28 to 40 with 3 to 15 years of job experience. Participants are chosen with the maximum diversity in sex, age and job experience.

#### 2.2.2 Data Collection and Analysis

The interview time was about 40 minutes, and each of the nurses was only interviewed once. The interviews were for nurses with at least 3 years of experience in the ICU. In this research, sampling was done from nurses to the point of data saturation. Some of the questions help were: “What’s your idea about the hope concept?” Does hope affect the way you take care of the patients? “or” Do you think your hope can affect the way other caregivers such as patient’s relatives or your colleagues take care of the patient?”

Conventional content analysis is simultaneously used with data collection. At first, after reading the implemented texts for several times and determining the unit of analysis, encoding process started. In the second phase of encoding, codes with similar meaning lied in one category. At first, primary codes were extracted. Finally, categories and sub - categories were determined through reduction classification process (Table 2). To determine data rigor, criteria such as dependability, credibility, transferability and confirmability are used (Lincoln & Guba, 1985). To determine dependability, findings are constantly compared. To determine credibility, the researcher was in touch with the research topic for a long time, and member check was used. To determine transferability, the findings were offered to 2 nurses who were not participating in the study but were qualified to. They verified the findings. To determine in the field work confirmability, constant use of interviews texts and notes in the field was used through incorporation of the findings.

**Table 2:** Attributes taken from the concept in field work phase

Category	Sub- category	Attributes	Confirming statements
Nursing care	-more comprehensive curing care - Continuing care	Promoting life quality, better function, more effort, spending more time, more effective care and better reporting	“Even in CPR which can be last care phase, it leads to keep on caring”.
Inner feeling	Pleasant feeling	Job pleasure, being motivated, feeling useful and spiritual.	
	Worry for the future	Satisfaction and burnout reduction	
Belief	Creating hope in other people.	Care accompanying hope, oriflamme, stating good experiences and play.	“Even in many cases, patients improve in the worst situation. These good experiences help us not lose our faith in further cases”. “When we hopefully care a patient, and show it in all my functions in the ICU, other colleagues better care the patients.”
	Despair		“Sometimes my hope is affected. For example, some people talk and say they work in the dead ward which has a bad effect on me.”
Outcome		Rebirth, improvement, Expect to improve, satisfy the needs, Death with dignity and positive experiences.	

### 2.3 Final Analytical Phase

Attributes taken from the field work phase are shown in Table 2. In theoretical phase, it was shown that most studies concentrated on the hope concept attributes from the viewpoint of the patient and his family. Nevertheless, nurse’s hope to patient’s improvement is a term having lots of functions in nursing discourse. Moreover, the hope concept affects the way the nursing care is presented. If we accept its role in promoting care quality, explaining its attributes will innately be of high importance. Merging the results of theoretical phase and field work phase, important attributes of the hope concept to patient’s improvement can be defined as a dynamic, realistic and goal- oriented expectation which offers a better function and attitude for offering effective nursing care accompanying nurse’s peace of mind. Generally, we can say that the hope concept to patient’s improvement is offering a comprehensive, and dynamic nursing care accompanying pleasant inner feeling affected by beliefs which has realistic consequences.

### 2.4 Ethics Consideration

Research license was taken from the Ethics Committee of Tehran University of Medical Sciences, and the researcher taking introduction letter from the Research Department started her study. Nurses consciously participated in the study, and they were assured that the information received is confidential. The time and the place of the interview were determined by the nurses.

## 3. Findings

The major findings of the study are shown in Table 2.

Some participants say about the nurse’s hope in patient’s improvement as following:

Nurse 4- “When I’m hopeful, I better perform the nursing care. I don’t want the patient to receive less care when he improves”.
Nurse 3- “Hope leads to more work and better function, because that time you think about what you want to achieve. For example, we expect patient’s improvement, so we perform our job with more care and higher quality”.
Nurse 1- “It has frequently happened that when my patient was in coma and suffered from loss of consciousness, I talked to him about his hopeful treatments. For example, I told him that some people came to visit him and who they were, or how well he responded to the treatment … Following me, all the treatment team did their best for his improvement.”
Nurse 6- “… Many patients were cared in the worst phases of their illness, and they improved. Well, these good experiences help the patient not lose his faith in further cases …”
Nurse 7- “… Hoping to improve patient means patient’s improvement and it helps a death with dignity in some cases.”
Nurse 4- “… When I’m hopeful, I feel satisfied, take pleasure from my job and do my job better.”
Nurse 2- “… When there is hope, you do any activity with more incentive and interest …. “
Nurse 5- “… When hopeful, you feel useful, satisfied with the job, motivated and spiritual.”

## 4. Discussion

In this study, some in the field work phase were consistent with the theoretical phase. One of them is better functioning or offering a better nursing care. Based on the studies, hope in nursing bears such as positive attitude, future- oriented, goal- oriented, realism, trying to improve, cognitive process, mobility, peace, and availability. In review of literature, some antecedents are presented for these specifications. In the previous study, power inside and positive feeling, for instance, are said to be antecedents of expectation and attaining the goal. Whereas, the present study showed that to have or to create hope does not necessarily mean to have such antecedents.

In fact, these two can be part of the hope, or they can be created while caring. Kylma et al. (2009) also considered hope as a power inside, while the participants did not directly talk about positive feeling or power inside in the data taken from the interviews. Instead, they considered it as a result of others’ perspectives and their own past experiences and believed them to create hope in nurses whose outcome was more trying and better functioning. One of the nurses participating in Reinke’s study (2010) who worked in Oncology said that nurses had a pivotal role in creating hope in patients and their families. If they appreciated this role, they would reinforce and better support the patient.

Another nurse attending in the same study declared, “… I appraised his hope every day and understood it could change. There is a delicate line between hope and denial; we walk on a blade which we clean it every day … (Reinke et al., 2010). In the present study, this aspect of hope is called as “belief”.

Another feature of the hope which is taken from the review of literature was nurse’s availability and being cognitive (Tutton et al., 2009; Klotz, 2010). Since offering nursing services requires knowledge and professional skill, and one of the hope antecedents in a nurse is to have professional skill whose result is a better, comprehensive and more effective nursing care, and since availability of a nurse is a pre- requisite for offering care and sustaining a high quality care, after this important feature of the hope concept in the nurse is used as “nursing care” in the present study.

O’Connor (1996) called hope to be not only a value but also as something necessary for health. Dufault and Martocchio (1985) believed hope to be action- oriented. People may do actions with hope which can directly affect the ideal result or attaining the hope. Another feature of hope taken from the review of literature is to make efforts for patient’s improvement and nurse’s peace: although it is called as hope feature in the review of literature, these two are, in fact, implicit consequences of hope in a nurse which is called hope feature.

In the present study, the participants believed this feature to be hope’s explicit consequences. In addition, Rusteon (1995) declared in his study although hope is determined through a future- oriented nature, it must be possible and expecting. If not, it shows a daily dream or escape mechanism. In fact, life satisfaction must be rooted in reality. A study was done by Penz (2008); he explained that one of the capabilities of hope in nurses, that is its outcome, is the effect on how they care of patients and also the effect of life quality on themselves and their patients. Self- efficacy was one of the other implicit features of hope taken from the review of literature (Gillespie et al., 2007). Self- efficacy implied the personal beliefs or judgments about one’s capabilities in doing duties and responsibilities (Bandura, 1997). In this study, a group of features such as job pleasure, being motivated, feeling useful, feeling spiritual, feeling satisfaction, and burnout reduction attained from the interviews are mentioned under three sub-categories: pleasant feeling, job satisfaction, and concern for the future and one major theme- “Feeling inside”. Rusteon (1995) called hope as a strategy for coping with tension which increases one’s compatibility. This strategy can reduce tension in nursing and give/offer job satisfaction.

### 4.1 Limitations

At last, the limitation of the present study was the lack of generalizability of the results due to non-probability sampling.

## 5. Conclusion

The findings of the present study showed that however the hope concept in nursing discourse has many implications; certain aspects of hope are concentrated in different people. Identifying four major themes taken from the interviews, it can be concluded that increasing nursing knowledge and knowing hope concepts can affect promoting more effective cares, pleasant inner feeling, creating positive attitude and expecting logical consequences such as improvement or death with dignity.

## Implications for Nursing Practice

With such an appreciation of the hope concept, nurses do their best in taking care of the patients.

## References

[ref1] Bandura A (1994). Self-efficacy.

[ref2] Benzein E, Saveman B. I (1998). One step towards the understanding of hope: a concept analysis. International Journal of Nursing Studies.

[ref3] Borimnejad L, Hamooleh M. M, Seyedfatemi N, Tahmasebi M (2014). Human Relationships in Palliative Care of Cancer Patient: Lived Experiences of Iranian Nurses. Materia socio-medica.

[ref4] Dufault K, Martocchio B. C (1985). Symposium on compassionate care and the dying experience. Hope: its spheres and dimensions. The Nursing Clinics of North America.

[ref5] Fitzgerald Miller J (2007). Hope: a construct central to nursing. In Nursing Forum.

[ref6] Gebhardt M. C, McGehee L. A, Grindel C. G, Testani-Dufour L (2011). Caregiver and nurse hopes for recovery of patients with acquired brain injury. Rehabilitation Nursing.

[ref7] Ghahremani Z, Alavi M. J, Hosseini F (2006). Correlates of quality of life in the family caregivers of schizophrenic patients with hope. Iran Journal of Nursing.

[ref8] Gillespie B. M, Chaboyer W, Wallis M (2007). Development of a theoretically derived model of resilience through concept analysis. Contemporary Nurse.

[ref9] Haase J. E, Britt T, Coward D. D, Leidy N. K, Penn P. E (1992). Simultaneous Concept Analysis of Spiritual Perspective, Hope, Acceptance and Self-transcendence. Image: The Journal of Nursing Scholarship.

[ref10] Klotz L. K (2010). Hope in relation to nursing interventions for HIV-infected patients and their significant others. Journal of the Association of Nurses in AIDS Care.

[ref11] Kylmä J, Duggleby W, Cooper D, Molander G (2009). Hope in palliative care: an integrative review. Palliative and supportive care.

[ref12] Lincoln Y. S (1985). Naturalistic inquiry.

[ref13] Meleis A. I (2011). Theoretical nursing: development and progress.

[ref14] Mok E, Lau K. P, Lam W. M, Chan L. N, Ng J, Chan K. S (2010). Health-care professionals’ perspective on hope in the palliative care setting. Journal of palliative medicine.

[ref15] O’Connor P (1996). Hope: a concept for home care nursing. Home care provider.

[ref16] Oxford Advanced Learner’s Dictionary (2010).

[ref17] Penz K (2008). Theories of hope: are they relevant for palliative care nurses and their practice?. International journal of palliative nursing.

[ref18] Reinke L. F, Shannon S. E, Engelberg R. A, Young J. P, Curtis J. R (2010). Supporting hope and prognostic information: nurses’ perspectives on their role when patients have life-limiting prognoses. Journal of pain and symptom management.

[ref19] Rustøen T (1995). Hope and quality of life, two central issues for cancer patients: a theoretical analysis. Cancer Nursing.

[ref20] Snyder C. R (1995). Conceptualizing, measuring, and nurturing hope. Journal of Counseling & Development.

[ref21] Tutton E, Seers K, Langstaff D (2009). An exploration of hope as a concept for nursing. Journal of Orthopaedic Nursing.

[ref22] (1995). Webster’s New World Dictionary.

